# Correlation of the Expression of BRAF V600E Mutation With Various Phenotypic Expressions of Thyroid Neoplasms

**DOI:** 10.7759/cureus.16048

**Published:** 2021-06-30

**Authors:** Volga Harikrishnan, Shantha Kumari, Subramaniam Ramkumar, Ramalingam Sankaran, Sudha Ramalingam, Thiagarajan Sairam

**Affiliations:** 1 Pathology, Saveetha Medical College, Chennai, IND; 2 Pathology, PSG institute of medical sciences and research, Coimbatore, IND; 3 Pathology, Woodland Hospital, Shillong, IND; 4 Pharmacology, PSG Institute of Medical Sciences and Research, Coimbatore, IND; 5 Community Medicine, PSG Institute of Medical Sciences and Research, Coimbatore, IND; 6 Molecular Biology, PSG Institute of Medical Sciences and Research, Coimbatore, IND

**Keywords:** braf v600e mutation, papillary carcinoma, thyroid gland, thyroid neoplasms, thyroid malignancies

## Abstract

Aims

We aimed to assess the incidence of the BRAF V600E mutation in thyroid neoplasms at a tertiary care center and its association with various phenotypic features.

Methods and material

We included all cases diagnosed as thyroid neoplasm in the past decade at the Department of Pathology of our institute and obtained their clinical details from the medical records department of the institute after obtaining permission from the authorities and due International Human Epigenome Consortium clearance. We included data on age, sex, clinical presentation, hormone status, and T and N status of the malignant neoplasms. Hematoxylin and eosin (H&E) slides of all cases were evaluated for the type of neoplasm, nuclear features, invasion into the capsule and vascular spaces, extrathyroidal extension, lymph node metastases, mitoses, necrosis, and presence/absence of amyloid. Paraffin blocks of sections with high tumor density and less normal tissue were chosen for evaluation after H&E staining. The slides showing tumors with large areas of hemorrhage, cystic change, or necrosis were excluded. Two primers were used to amplify a 339-bp fragment containing the V600E mutation in exon 15 of BRAF. Tissues were prepared from formalin-fixed paraffin-embedded (FFPE) blocks, and DNA was isolated using a standard protocol BRAF NF and BRAF NR Primer Standardized Protocol For FFPE Tissue DNA. Percentages and tables have been used for data presentation.

Results

Among 47 identified cases, 14 were positive for the BRAF V600E mutation and had papillary carcinoma (n = 9) or follicular neoplasms (n = 5; follicular adenoma, n = 3; follicular carcinoma, n = 2). In the BRAF-positive papillary carcinomas, five cases were aged 20-30 years, eight were female, eight (88.88%) were euthyroid, and one was hypothyroid. Furthermore, 55.55% (5/9 cases) of BRAF-positive cases were stage I, 33.3% (3/9 cases) were stage II, and 0.02% (1/9 cases) were stage III.

Conclusions

In our cohort, 31% of cases of papillary thyroid carcinoma (PTC) and 18.72% of follicular neoplasms expressed the BRAF V600E mutation.

BRAF V600E mutation-positive papillary thyroid carcinomas consistently showed all characteristic nuclear features, such as nuclear crowding, overlapping, and grooves.

Considering the greater prevalence in the younger age group, the importance of mutation surveillance in PTCs for a total thyroidectomy may be warranted in mutation-positive patients.

## Introduction

Thyroid cancers account for approximately 1% of all malignancies and are one of the most common malignancies of the endocrine system [1,2]. The development of malignancy in the thyroid is influenced by both environmental factors as well as genetic factors. Specifically, the thyroid is vulnerable to genotoxic effects of radioactive iodine and nongenotoxic effects (thyroid-stimulating hormone stimulation) of iodine deficiency both of which can lead to neoplastic lesions in the thyroid [3].

Most thyroid tumors can be diagnosed based on morphological assessment and immunohistochemical findings [4,5] As altered gene expression can be discovered before morphological changes, knowledge regarding the genetic background helps in understanding thyroid carcinogenesis and facilitates the planning of appropriate therapeutic strategies and development of drugs that target specific molecules.

Thyroid tumors are classified based on the 2004 World Health Organization guidelines [3]. Recently, the RAS-RAF-MAP kinase signaling pathway has been implicated in many cancers, particularly more frequently in thyroid carcinomas, with the BRAF V600E mutation playing a vital role in papillary thyroid carcinoma. Furthermore, the BRAF mutation is specific for papillary carcinoma, is associated with poorer outcomes, and is frequently noted in high-grade tumors [6-8]

BRAF: an overview

BRAF is a serine-threonine kinase belonging to the family of RAF proteins. It acts upstream of MEK1/2 kinases in response to RAS signals. BRAF mutations are involved in early thyroid carcinogenesis and are somatic genetic alterations rather than germline mutations [9,10]. BRAF mutations occur early and play an important role in the pathogenesis of papillary thyroid carcinoma (PTC). Point mutations of BRAF are the most common types of mutations and occur in approximately 40%-45% of cases [11,12]. Other studies have reported [13,14] BRAF mutation rates of 35%-69% in PTC. The most common BRAF mutation is V600E, which is from missense thymine (T) to adenine (A) transversion at nucleotide 1799 in exon 15. This results in the substitution of valine by glutamate at residue 600. A less common mutation is the K601E mutation found in thyroid cancer [15,16]. Structurally, the RAF protein is divided into two functional domains, namely, the N-terminal and C-terminal regulatory domains, with three conserved regions (CR 1,2, and 3). CR1 and CR2 are present in the N-terminal domain and CR3 in the C-terminal domain [17].

Normally, RAS proteins are attached to the plasma membrane on the cytoplasmic side of the cell, endoplasmic reticulum, and Golgi membrane and are activated by growth factors binding to receptors at the plasma membrane. In an inactive state, RAS proteins bind to guanosine diphosphate (GDP), and upon activation, exchange GDP with guanosine triphosphate (GTP), leading to a conformational change that produces active RAS [2]. This activated RAS binds to the RAS-binding domain in CR1 of RAF, recruits RAF to the membrane, and activates a downstream signaling cascade wherein RAF phosphorylates mitogen-activated protein kinase (MAPK) [17]. Extracellular signal-regulated kinases 1 and 2 (MEK 1 and 2) become activated, which consecutively phosphorylate and activate extracellular signal-regulated kinases 1 and 2 (ERK 1 and 2). Activated ERK migrates to the nucleus where it activates various transcription factors that trigger cell cycle progression and result in cell proliferation and differentiation [8]. The RAF protein has three isoforms, ARAF, BRAF, and CRAF. Among these, BRAF is the most common isoform and most commonly mutated isoform because it has higher basal kinase activity [17] Additionally, BRAF protein expression is higher in hematopoietic cells, neurons, and testicles; is the predominant isoform [18,19] in thyroid follicular cells; and is the most potent activator of the MAPK pathway. Gain-of-function BRAF mutations function as an alternate pathway for aberrant activation of ERK signaling, resulting in constitutive activation of BRAF kinase, i.e., it can phosphorylate MEK as monomers in a RAS-independent manner. Such chronic stimulation of the MAPK pathway results in increased proliferation but reduced survival and differentiation of cells [11]. This signaling cascade is implicated in the etiology of several human cancers including malignant melanoma; thyroid, colorectal, and ovarian carcinomas; and carcinomas of the biliary tract, ovary, and cervix [9,20]

BRAF expression in thyroid carcinomas

Thyroid malignancies are predominantly diagnosed based on morphological assessment alone, and an immunohistochemical study is beneficial in confirming the diagnosis and establishing prognosis if tumor(s) exhibits unusual patterns. A significant improvement has been noted in the knowledge of molecular alterations over the past two decades in all tumors, including thyroid malignancies, and oncogenic BRAF activation represents one of the most prevalent molecular alterations. Reportedly, BRAF mutations are associated with poorer clinicopathological outcomes [21,22]. Among its various subtypes, BRAF most commonly occurred in the tall cell variant of PTC, followed by conventional PTC, and less commonly in the follicular variant of PTC. The tall cell variant is the most aggressive tumor, indicating that BRAF mutations are associated with poorer outcomes [9,23]. Other factors associated with the mutation and aggressive tumor phenotype include older age, extrathyroidal tumor invasion, lymph node, and distant metastasis, higher tumor stage, and poorly differentiated cancer [21,22,24-26].

Frasca et al [27] found that the presence of the BRAF V600E mutation in PTC is associated with aggressive tumor behavior and that tumor aggressiveness is independent of tumor size, suggesting that even small BRAF-positive tumors carry a higher risk of progression and invasiveness than BRAF-negative tumors.

BRAF mutations can be readily tested on thyroid tissue samples obtained by fine-needle aspiration with high preoperative clinicopathological prognostic capabilities in PTC. However, they have limited diagnostic value because [28-30] of low sensitivity in cytologically indeterminate specimens that are mostly non-PTC and therefore do not harbor the BRAF mutation. Nonetheless, controversies regarding BRAF mutations with poorer clinicopathological outcomes in papillary thyroid cancers have been reported in some studies [31,32]. In the thyroid, apart from papillary carcinomas, BRAF mutations are expressed in anaplastic carcinoma and poorly differentiated carcinoma (prevalence, 20%-30%, and 10%-15%, respectively). 

BRAF-mutated, poorly differentiated, anaplastic carcinoma will have a papillary component, suggesting that these tumors progress from BRAF-positive papillary carcinoma [33-36]. The BRAF V600E mutation is not found in follicular thyroid cancers and benign thyroid nodules [23]. However, the other BRAF mutation, K601E, has been detected in follicular adenoma, carcinoma, and follicular variants of PTC. However, Kebebew et al reported a BRAF V600E mutation in one case of follicular carcinoma [22]. Newly developed mitogen extracellular kinase (MEK) inhibitors are particularly promising therapeutic agents for thyroid cancer. With these advances, it has become clearer that BRAF mutation will likely have a significant impact on the clinical management of PTC. Thus, this study aimed to assess the incidence of the BRAF V600E mutation in thyroid neoplasms at a tertiary care center and its association with various phenotypic features.

## Materials and methods

**Figure 1:**
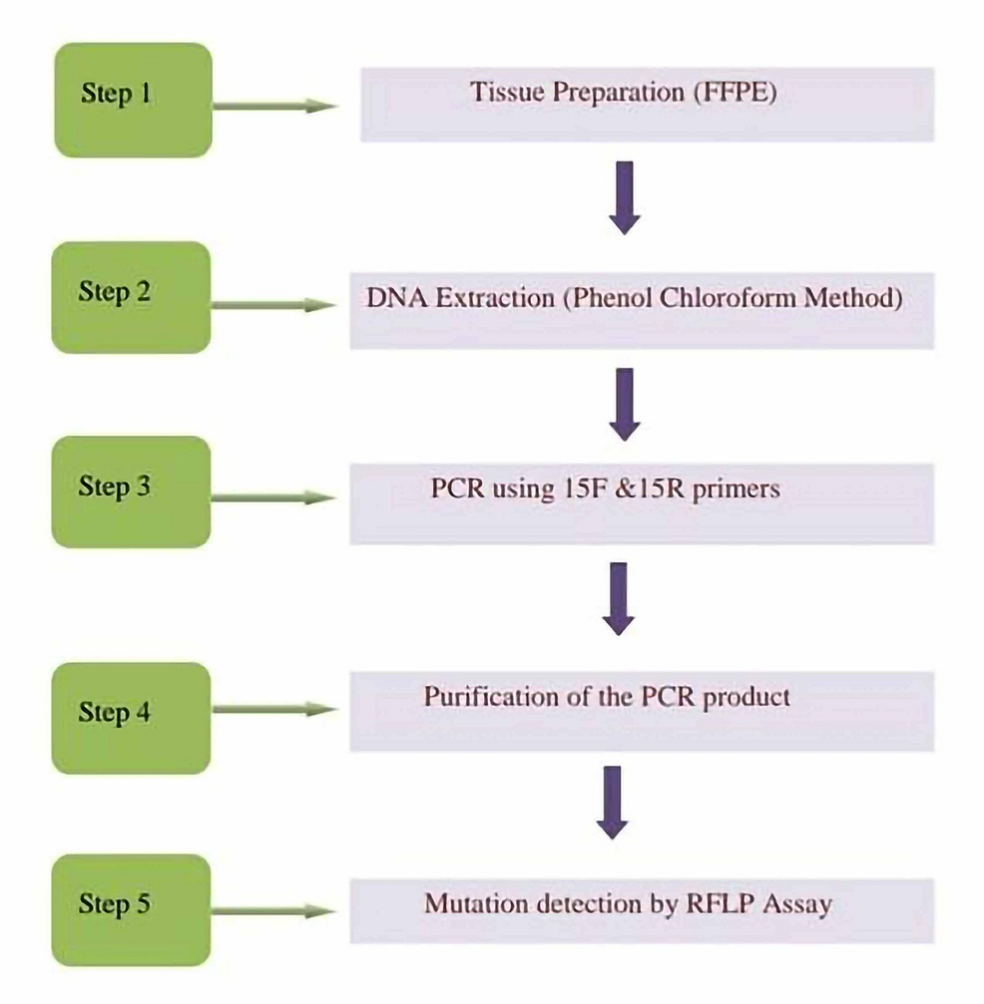
Steps in BRAF mutation analysis in various thyroid neoplasms. FFPE: formalin-fixed paraffin-embedded; PCR: polymerase chain reaction; RFLP: restriction fragment length polymorphism

**Figure 2:**
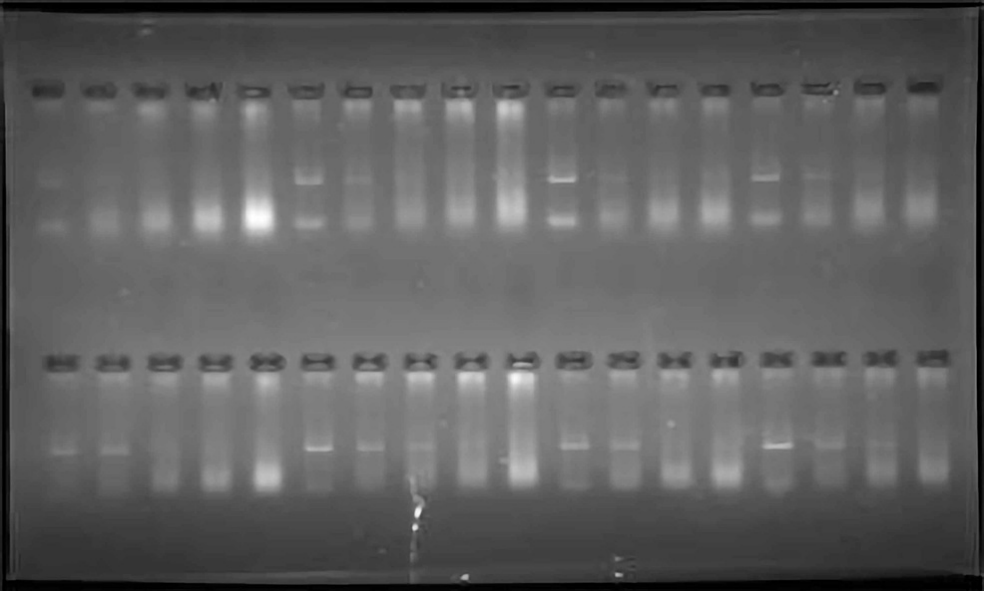
Agarose gel electrophoresis: DNA extraction.

**Figure 3:**
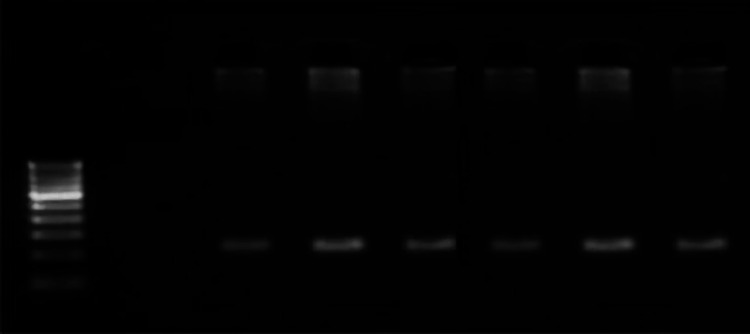
Agarose gel electrophoresis: PCR amplification. PCR: polymerase chain reaction

**Figure 4:**
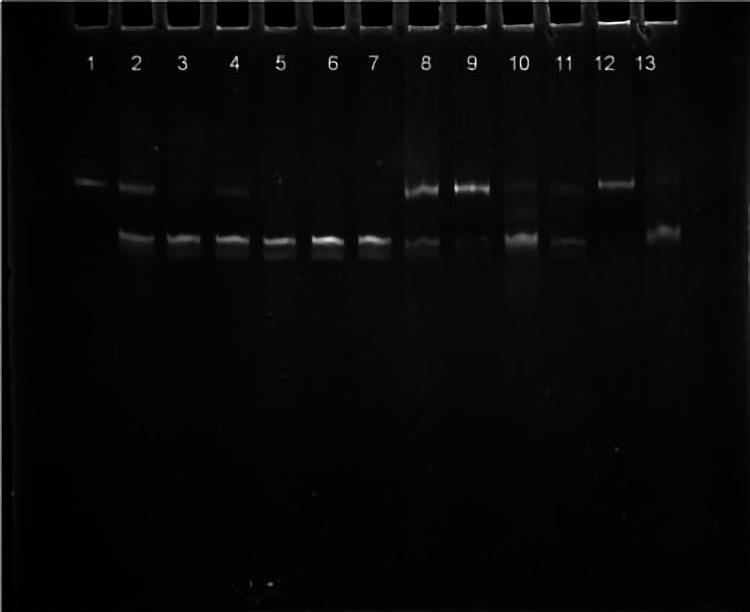
Polyacrylamide gel electrophoresis: restriction digestion.

We included all cases diagnosed as thyroid neoplasm in the past decade at the Department of Pathology of our institute and obtained their clinical details from the medical records department of the institute after obtaining permission from the authorities and due International Human Epigenome Consortium clearance (IRB number/Proposal number: 10/098). We included data on age, sex, clinical presentation, hormone status, and T and N status of the malignant neoplasms. Hematoxylin and eosin (H&E) slides of all cases were evaluated for the type of neoplasm, nuclear features, invasion into the capsule and vascular spaces, extrathyroidal extension, lymph node metastases, mitoses, necrosis, and presence/absence of amyloid. Paraffin blocks of sections with high tumor density and less normal tissue were chosen for evaluation after H&E staining. The slides showing tumors with large areas of hemorrhage, cystic change, or necrosis were excluded. Two primers were used to amplify a 339-bp fragment containing the V600E mutation in exon 15 of BRAF. Tissues were prepared from formalin-fixed paraffin-embedded (FFPE) blocks, and DNA was isolated using a standard protocol BRAF NF & BRAF NR Primer Standardized Protocol For FFPE Tissue DNA (Figures 1-4). Percentages and tables have been used for data presentation.

## Results

Of the 50 identified cases, DNA could only be extracted from 47 because extraction proved futile in the remaining three cases (Table 1).

**Table 1:** Master chart showing demographic variables, morphologic subtype of thyroid neoplasms, stage, and mutant status. PTC: papillary thyroid carcinoma; FN-LMP: follicular neoplasm - low malignant potential

S.No	Age/ Sex	Hormone Status	Size	Other features	Diagnosis	Stage	BRAF
1	48/F	Euthyroid	0.4cm	-	Papillary microcarcinoma	I	Mutant
2	65/F	Hypothyroid	1.3cm	-	Papillary carcinoma	I	Mutant
3	30/F	Euthyroid	0.3cm	-	Encapsulated follicular variant of Papillary carcinoma	I	Mutant
4	28/F	Euthyroid	0.3, 1.4cm	Multifocal, cystic	Papillary carcinoma	I	Mutant
5	32/F	Euthyroid	1cm	Cystic	Papillary carcinoma	I	Mutant
6	48/M	NA	4.2cm	-	Follicular variant of PTC	I	Wild
7	38/M	Euthyroid	0.5cm	-	Papillary microcarcinoma	I	Wild
8	50/F	Euthyroid	0.3- 1.0cm	Multifocal	Papillary carcinoma	I	Wild
9	30/F	Euthyroid	0.4cm	-	Papillary microcarcinoma	I	Wild
10	53/M	Euthyroid	2.0cm	-	Papillary carcinoma	I	Wild
11	27/F	Hyperthyroid	0.5cm	-	Papillary microcarcinoma	I	Wild
12	32/F	Euthyroid	1.5cm	-	Papillary carcinoma	I	Wild
13	41/F	Euthyroid	0.3-0.8cm	Multifocal, stromal bone formation, FA	Papillary carcinoma	I	Wild
14	52/F	Euthyroid	0.4- 2.3cm	Multifocal	Papillary carcinoma	II	Mutant
15	29/M	Euthyroid	2.5cm	-	Papillary carcinoma	II	Mutant
16	27/F	Euthyroid	2.4cm	-	Papillary carcinoma	II	Mutant
17	49/F	Euthyroid	3.5cm	Multifocal, cystic	Papillary carcinoma	II	Wild
18	51/F	Euthyroid	3cm	Cystic	Papillary carcinoma	II	Wild
19	70/M	Euthyroid	2.0- 4.0cm	Multifocal	Papillary carcinoma	II	Wild
20	27/F	Euthyroid	3.0cm	-	Papillary carcinoma	II	Wild
21	45/F	Euthyroid	3.5cm	-	Papillary carcinoma	II	Wild
22	59/M	Euthyroid	1.0-3.0cm	Multifocal	Papillary carcinoma	II	Wild
23	20/F	Euthyroid	3.5cm	Multifocal, capsular and lymph node involvement	Papillary carcinoma	III	Mutant
24	50/F	Euthyroid	NA	Extrathyroidal extension	Papillary carcinoma	III	Wild
25	23/F	Euthyroid	5.5cm	-	Papillary carcinoma	III	Wild
26	46/F	Euthyroid	0.8- 5.0cm	Multifocal	Papillary carcinoma	III	Wild
27	24/F	Euthyroid	5.5cm	Cystic	Papillary carcinoma	III	Wild
28	50/M	Euthyroid	2.0cm	Extrathyroidal extension	Papillary carcinoma	III	Wild
29	45/F	NA	3.5cm	lymph node involvement, FA	Papillary carcinoma	III	Wild
30	43/M	Euthyroid	2.5cm	-	Follicular adenoma	-	Mutant
31	58/M	Euthyroid	7.0cm	-	Microfollicular adenoma- signet ring cell type	-	Mutant
32	35/F	Euthyroid	4.0cm	-	Follicular adenoma	-	Mutant
33	32/F	Euthyroid	2.3cm	-	Follicular adenoma	-	Wild
34	35/F	Euthyroid	9.5cm	-	Follicular adenoma	-	Wild
35	34/F	Euthyroid	3.0cm	-	Follicular adenoma	-	Wild
36	43/F	Euthyroid	3.3cm	-	Follicular adenoma	-	Wild
37	36/F	Euthyroid	5.5cm	-	Follicular adenoma	-	Wild
38	45/F	Euthyroid	2.5cm	-	Follicular adenoma	-	Wild
39	34/F	Euthyroid	3.0cm	-	Follicular adenoma	-	Wild
40	35/F	Hyperthyroid	4.5cm	-	Follicular adenoma	-	Wild
41	55/F	Euthyroid	1.0cm	-	Follicular adenoma	-	Wild
42	30/M	Euthyroid	2.6cm	-	Minimally invasive follicular carcinoma	-	Mutant
43	45/F	Euthyroid	6.0cm	-	Hurthle cell neo cap	-	Mutant
44	57/M	Euthyroid	3.0cm	-	FN-LMP	-	Wild
45	30/F	Hypothyroid	3.0cm	-	Minimally invasive follicular carcinoma	-	Wild
46	38/M	Euthyroid	1.8cm	-	Medullary carcinoma	IV A	Wild
47	40/F	Euthyroid	11.5cm	Follicular carcinoma	Anaplastic carcinoma	IVB	Wild

**Figure 5:**
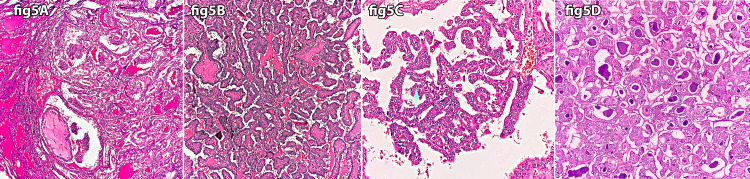
Papillary thyroid carcinoma H&E stain (A x10, B x20, C x20, D x20). A, B, C: Papillary thyroid carcinoma - Showing characteristic papillary architecture, characteristic nuclear features with ground class chromatin, nuclear grooving, nuclear crowding, nuclear overlapping, and intranuclear cytoplasmic pseudo-inclusions. D: Papillary thyroid carcinoma (follicular variant) - Showing prominent follicular architecture with characteristic nuclear features with ground class chromatin, nuclear grooving, nuclear crowding, nuclear overlapping, and intranuclear cytoplasmic pseudo-inclusions.

Only 14 cases were positive for the BRAF mutation; of these, nine were of papillary carcinoma (Figure 5).

**Figure 6:**
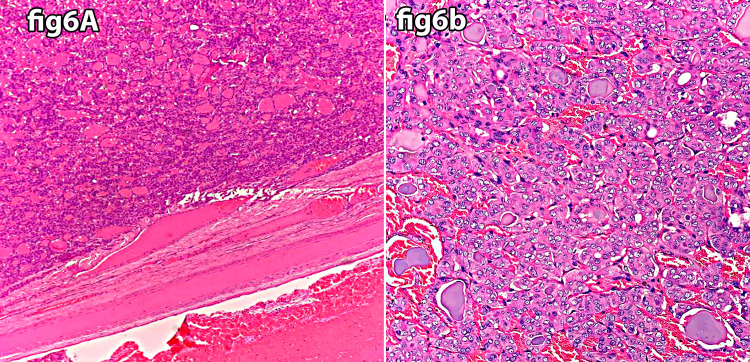
Follicular adenoma H&E stain (A x20, B x40). A: Follicular adenoma showing marked circumscription and encapsulation. B: Follicular adenoma showing a prominent repetitive microfollicular pattern.

Out of the 14 BRAF-positive cases, five were follicular neoplasms, with the latter category comprising three cases of follicular adenomas and two of follicular carcinomas (Tables 1, 2 ) (Figure 6).

**Table 2:** Break up of BRAF V600E-positive cases (n = 47).

Types of neoplasms	BRAF V600E positive - mutant type (%)	Wild type (%)	Total
Papillary carcinoma: (Conventional papillary thyroid carcinoma - 8 cases) (Encapsulated follicular variant of papillary thyroid carcinoma - 1 case)	9 (31%)	20 (69%)	29
Follicular neoplasm: (Follicular adenoma - 3 cases) (Follicular carcinoma - 2 cases)	5 (31.25%)	22 (68.75%)	16
Medullary carcinoma	0	1 (100%)	1
Anaplastic carcinoma	0	1 (100%)	1
Total	14 (29.8%)	33 (70.2%)	47

The nine cases of BRAF-positive papillary carcinomas predominantly comprised individuals aged 20-30 years (Tables 1, 3), were almost all female (8/9) (Tables 1, 4), and were euthyroid (8/9, 88.88%) (Tables 1, 5).

**Table 3:** Number of BRAF-positive papillary thyroid carcinoma and age distribution.

Age group	Number of cases
20–30 years	5
31–40 years	1
41–50 years	1
51–60 years	1
61–70 years	1

**Table 4:** BRAF-positive carcinomas and sex distribution.

Male	Female
One case	Eight cases

**Table 5:** BRAF-positive papillary thyroid carcinoma and hormonal status.

Number of BRAF-positive cases	Hormonal status
8	Euthyroid
1	Hypothyroid

Next, eight of the nine cases were euthyroid (88.88%), whereas one was hypothyroid (Tables 1, 5). Additionally, 55.55% of BRAF V600E-positive cases were found to be stage I (5/9 cases), 33.33% to be stage II (3/9 cases), and 11.11% to be stage III (1/9 cases) (Tables 1, 6).

**Table 6:** BRAF-positive papillary thyroid carcinoma stage at presentation.

BRAF-positive papillary thyroid carcinoma staging	Number of cases
Stage I	5
Stage II	3
Stage III	1
Stage IV	0

Scoring for nuclear and other microscopic features revealed characteristic features compared with other features observed for BRAF-positive papillary carcinomas (Tables 7, 8). The scoring was done on a relative qualitative double consensus scale based on mild (Score 1), moderate (Score 2), and severe (Score 3) appearance of the nuclear features. 

**Table 7:** Relative qualitative double consensus scoring for nuclear features of papillary thyroid carcinoma.

Histologic nuclear feature	Mild	Moderate	Severe
Nuclear enlargement (NE)	1	2	3
Nuclear irregularity (NI)	1	2	3
Chromatin clearing (CC)	1	2	3
Nuclear crowding (NC)	1	2	3
Nuclear grooves (NG)	1	2	3
Nuclear pseudoinclusions (PI)	1	2	3
Fibrosis (Fib)	1	2	3
Psammoma bodies (PB)	1	2	3

**Table 8:** Scoring for nuclear features and other microscopic features. NE: nuclear enlargement; NI: nuclear irregularity; CC: chromatin clearing; NC: nuclear crowding; NG: nuclear grooves; PI: nuclear pseudoinclusion; Fib: fibrosis; PB: psammoma bodies; HPF: high-power fields

S. No.	Id. No.	NE	NI	CC	NC	NG	PI/10HPF	Fib	PB/10HPF	Inflammatory cell aggregate/10HPF
1	458/06	1	1	2	3	3	0	1	2	1
2	2780/06	2	1	3	3	2	0	0	0	0
3	2874/07	2	1	3	3	2	1	1	0	0
4	1837/08	2	2	1	3	2	2	1	0	0
5	1865/08	2	1	3	2	2	1	1	0	0
6	2222/08	2	1	3	2	1	0	2	2	0
7	229/09	2	0	1	3	2	0	0	0	0
8	1218/09	2	0	1	2	3	0	0	0	0
9	2548/09	3	3	1	1	2	2	3	3	3
Average score		2	1.1	2	2.4	2.1	0.6	1	0.7	0.4

## Discussion

The BRAF V600E mutation is most often expressed in stage III (30%). Importantly, its expression, particularly in the early stages, indicates an aggressive course and warrants a total thyroidectomy, whereas its expression in the later stages is associated with poor outcomes.

In our group of 30 papillary carcinoma cases, DNA could be extracted from 29 and only nine expressed the BRAF V600E mutation. Its prevalence is lower than that reported in the literature, i.e., 40%-45% of PTCs harbor the mutation.

We found that five of the nine cases of BRAF V600E-positive papillary carcinoma were in the 20-30-year age group. Rossella et al found that V600E was expressed more in an older age group [6]. Given the greater prevalence in the younger age group, the importance of mutation surveillance in PTCs for a total thyroidectomy may be warranted in mutation-positive patients.

We found five cases of stage I (55.55%) and three cases of stage II (33.33%) showing BRAF positivity, indicating a significantly higher expression in stages I and II lesions. Therefore, we reanalyzed the reports of PTC.

This high incidence of V600E mutation can be explained by the fact that at a tertiary care hospital, the radiologist and cytopathologist could probably detect PTC at an early stage. Furthermore, the strongest argument against using BRAF mutation as an independent prognostic and predictive factor in PTC is its high prevalence (30%-80%). As the V600E mutation is associated with poor outcomes, these patients require very close follow-up. We found no correlation between nuclear features and BRAF V600E mutations; however, the characteristic nuclear crowding, overlapping, and grooves were consistently observed (Tables 6, 7).

The incidence of the BRAF V600E mutation in follicular neoplasms was 31% (five out of 16 cases ). In contrast, the literature shows that the V600E mutation is rarely seen in follicular neoplasms, with very few reports that have identified this mutation in follicular neoplasms with minimal incidence [37]. Therefore, a reanalysis of routine H&E slides identified two cases of focal papillary architecture with nuclear features, which might have been overlooked during the morphological assessment. Despite their exclusion, the percentage incidence of the V600E mutation in follicular neoplasm was 18.72% (three out of 16 cases). Nonetheless, papillary carcinoma, particularly the microfollicular type, was possibly missed on routine evaluation as specimens were not extensively sampled, rather were only drawn from the most representative areas of the lesion.

Overall, we show that a significant number of cases of papillary carcinoma (31%; 9/29) and follicular neoplasms (18.7%; 3/16) express the V600E mutation. A larger prospective study, including preoperative cytology specimen and surgically resected specimens of the same patients, is needed to establish the utility of the V600E mutation as a routine diagnostic tool. After development, the primers would be cost-effective to patients.

An increase in the incidence of papillary carcinoma in areas where there is high iodine intake has been reported [3,38,39] and there is high iodine supplementation to correct iodine deficiency, particularly in the population with an existing V600E mutation. This raises the question of establishing dietary iodine supplementation levels to prevent the incidence of non-neoplastic conditions including multinodular goiter. Furthermore, papillary carcinoma usually remains intra-thyroid and few cases may present with rapid onset of clinical manifestations with enlargement of multiple cervical lymph nodes [40]. Therefore, a larger, multicenter, prospective study that includes cytology specimens collected preoperatively and following surgical resection is essential to establish the relationship between the incidence of papillary carcinoma and V600E mutation in the background of iodine supplementation and deficiency and to prove the utility of BRAF in routine diagnosis. However, the most recent American Thyroid Association (ATA) recommendations do not indicate a routine application of BRAF status for initial risk stratification in differentiated thyroid cancer due to a lack of evident confirmation of a direct influence of mutation on the increase in relapse risk.

Further, considering the increase in the prevalence of the BRAF V600E mutation in follicular neoplasms, a larger study of the V600E mutation in follicular neoplasms is vital to observe changes in the expression patterns of mutations in the Indian population.

## Conclusions

In our cohort, 31% of cases of PTC and 18.72% of follicular neoplasms expressed the BRAF V600E mutation.

BRAF V600E mutation-positive PTCs consistently showed all characteristic nuclear features, such as nuclear crowding, overlapping, and grooves.

Considering the greater prevalence in the younger age group, the importance of mutation surveillance in PTCs for a total thyroidectomy may be warranted in mutation-positive patients.

## References

[1] Carcangiu ML (2006). Thyroid gland. Histology for Pathologists, Third Edition.

[2] DeLellis R, Lloyd RV, Heitz PU, Eng C (2004). World Health Organization Classification of Tumours: Pathology and Genetics of Tumours of Endocrine Organs. IARC Press.

[3] Abul K, Abbas AM (2004). The endocrine system. Robbins and Cotran Pathological Basis of Disease.

[4] Cheung CC, Ezzat S, Freeman JL, Rosen IB, Asa SL (2001). Immunohistochemical diagnosis of papillary thyroid carcinoma. Mod Pathol.

[5] Tastekin E, Keskin E, Can N (2019). CD56, CD57, HBME1, CK19, Galectin-3 and p63 immunohistochemical stains in differentiating diagnosis of thyroid benign/malign lesions and NIFTP. Pol J Pathol.

[6] Elisei R, Ugolini C, Viola D (2008). BRAF(V600E) mutation and outcome of patients with papillary thyroid carcinoma: a 15-year median follow-up study. J Clin Endocrinol Metab.

[7] Al-Salam S, Sharma C, Afandi B, Al Dahmani K, Al-Zahrani AS, Al Shamsi A, Al Kaabi J (2020). BRAF and KRAS mutations in papillary thyroid carcinoma in the United Arab Emirates. PLoS One.

[8] Al-Brahim N, Asa SL (2006). Papillary thyroid carcinoma: an overview. Arch Pathol Lab Med.

[9] Xing M (2007). BRAF mutation in papillary thyroid cancer: pathogenic role, molecular bases, and clinical implications. Endocr Rev.

[10] Singh A, Ham J, Po JW, Niles N, Roberts T, Lee CS (2021). The genomic landscape of thyroid cancer tumourigenesis and implications for immunotherapy. Cells.

[11] Nikiforov YE (2011). Molecular diagnostics of thyroid tumors. Arch Pathol Lab Med.

[12] Qi W, Shi C, Zhang P, Feng L, Wang J, Chen D (2020). Effect of BRAF V600E mutation detection of fine-needle aspiration biopsy on diagnosis and treatment guidance of papillary thyroid carcinoma. Pathol Res Pract.

[13] Kato MA, Fahey TJ 3rd (2009). Molecular markers in thyroid cancer diagnostics. Surg Clin North Am.

[14] Ferrari SM, Fallahi P, Ruffilli I (2018). Molecular testing in the diagnosis of differentiated thyroid carcinomas. Gland Surg.

[15] Patel KN, Singh B (2006). Genetic considerations in thyroid cancer. Cancer Control.

[16] Giordano TJ (2018). Genomic hallmarks of thyroid neoplasia. Annu Rev Pathol.

[17] Vakiani E, Solit DB (2011). KRAS and BRAF: drug targets and predictive biomarkers. J Pathol.

[18] Al-Humadi H, Zarros A, Al-Saigh R, Liapi C (2010). Genetic basis and gene therapy trials for thyroid cancer. Cancer Genomics Proteomics.

[19] Laetitia G, Sven S, Fabrice J (2020). Combinatorial therapies in thyroid cancer: an overview of preclinical and clinical progresses. Cells.

[20] Wan PTC, Garnett MJ, Roe SM (2004). Mechanism of activation of the RAF-ERK signaling pathway by oncogenic mutations of B-RAF. Cell.

[21] Scheffel RS, de Cristo AP, Romitti M (2021). The BRAFV600E mutation analysis and risk stratification in papillary thyroid carcinoma. Arch Endocrinol Metab.

[22] Kebebew E, Weng J, Bauer J (2007). The prevalence and prognostic value of BRAF mutation in thyroid cancer. Ann Surg.

[23] Kondo T, Ezzat S, Asa SL (2006). Pathogenetic mechanisms in thyroid follicular-cell neoplasia. Nat Rev Cancer.

[24] Nakayama H, Yoshida A, Nakamura Y (2007). Clinical significance of BRAF (V600E) mutation and Ki-67 labeling index in papillary thyroid carcinomas. Anticancer Res.

[25] Chen P, Pan L, Huang W (2020). BRAF V600E and lymph node metastases in papillary thyroid cancer. Endocr Connect.

[26] Espenbetova M, Krykpayeva A, Zamanbekova Z, Zhumanbaeva Z, Azizov B, Mukanova A, Glushkova N (2021). Analysis of the association of BRAFV600E mutation and Ki-67 overexpression with clinical and pathological characteristics in papillary thyroid cancer. Radiat Environ Biophys.

[27] Frasca F, Nucera C, Pellegriti G (2008). BRAF(V600E) mutation and the biology of papillary thyroid cancer. Endocr Relat Cancer.

[28] Rowe LR, Bentz BG, Bentz JS (2006). Utility of BRAF V600E mutation detection in cytologically indeterminate thyroid nodules. Cytojournal.

[29] Zhao CK, Zheng JY, Sun LP, Xu RY, Wei Q, Xu HX (2019). BRAFV600E mutation analysis in fine-needle aspiration cytology specimens for diagnosis of thyroid nodules: the influence of false-positive and false-negative results. Cancer Med.

[30] Paek SH, Kim BS, Kang KH, Kim HS (2017). False-negative BRAF V600E mutation results on fine-needle aspiration cytology of papillary thyroid carcinoma. World J Surg Oncol.

[31] Sapio MR, Posca D, Troncone G (2006). Detection of BRAF mutation in thyroid papillary carcinomas by mutant allele-specific PCR amplification (MASA). Eur J Endocrinol.

[32] Costa AM, Herrero A, Fresno MF, Heymann J, Alvarez JA, Cameselle-Teijeiro J, García-Rostán G (2008). BRAF mutation associated with other genetic events identifies a subset of aggressive papillary thyroid carcinoma. Clin Endocrinol (Oxf).

[33] DeLellis RA (2006). Pathology and genetics of thyroid carcinoma. J Surg Oncol.

[34] Volante M, Lam AK, Papotti M, Tallini G (2021). Molecular pathology of poorly differentiated and anaplastic thyroid cancer: what do pathologists need to know?. Endocr Pathol.

[35] Odate T, Oishi N, Kawai M (2021). Progression of papillary thyroid carcinoma to anaplastic carcinoma in metastatic lymph nodes: solid/insular growth and hobnail cell change in lymph nodes are predictors of subsequent anaplastic transformation. Endocr Pathol.

[36] Quiros RM, Ding HG, Gattuso P, Prinz RA, Xu X (2005). Evidence that one subset of anaplastic thyroid carcinomas are derived from papillary carcinomas due to BRAF and p53 mutations. Cancer.

[37] Soares P, Trovisco V, Rocha AS (2003). BRAF mutations and RET/PTC rearrangements are alternative events in the etiopathogenesis of PTC. Oncogene.

[38] Harach HR, Escalante DA, Day ES (2002). Thyroid cancer and thyroiditis in Salta, Argentina: a 40-yr study in relation to iodine prophylaxis. Endocr Pathol.

[39] Harach HR, Ceballos GA (2008). Thyroid cancer, thyroiditis and dietary iodine: a review based on the Salta, Argentina model. Endocr Pathol.

[40] Jagtap SV, Patil D, Chetan Chetan, Gupta SO (2018). Papillary carcinoma thyroid presented with extensive local lymph nodal metastasis. P Arch Cytol Histopathol Res.

